# Recommendations for daytime, evening, and nighttime indoor light exposure to best support physiology, sleep, and wakefulness in healthy adults

**DOI:** 10.1371/journal.pbio.3001571

**Published:** 2022-03-17

**Authors:** Timothy M. Brown, George C. Brainard, Christian Cajochen, Charles A. Czeisler, John P. Hanifin, Steven W. Lockley, Robert J. Lucas, Mirjam Münch, John B. O’Hagan, Stuart N. Peirson, Luke L. A. Price, Till Roenneberg, Luc J. M. Schlangen, Debra J. Skene, Manuel Spitschan, Céline Vetter, Phyllis C. Zee, Kenneth P. Wright

**Affiliations:** 1 Centre for Biological Timing, Faculty of Biology, Medicine and Health, University of Manchester, Manchester, United Kingdom; 2 Department of Neurology, Thomas Jefferson University, Philadelphia, Pennsylvania, United States of America; 3 Centre for Chronobiology, University Psychiatric Clinics Basel, Transfaculty Research Platform Molecular and Cognitive Neurosciences, University of Basel, Basel, Switzerland; 4 Division of Sleep and Circadian Disorders, Departments of Medicine and Neurology, Brigham and Women’s Hospital, Boston, Massachusetts, United States of America; 5 Division of Sleep Medicine, Harvard Medical School, Boston, Massachusetts, United States of America; 6 Surrey Sleep Research Centre, Faculty of Health and Medical Sciences, University of Surrey, Guildford, United Kingdom; 7 Research Centre for Hauora and Health, Massey University, Wellington, New Zealand; 8 Centre for Radiation, Chemical and Environmental Hazards, Public Health England, Chilton, Didcot, United Kingdom; 9 Sleep and Circadian Neuroscience Institute, Nuffield Department of Clinical Neurosciences, University of Oxford, Oxford, United Kingdom; 10 Institutes for Medical Psychology and Occupational, Social and Environmental Medicine, Medical Faculty, Ludwig-Maximilians University (LMU), Munich, Germany; 11 Human Technology Interaction Group, Department of Industrial Engineering and Innovation Sciences, Eindhoven University of Technology, Eindhoven, the Netherlands; 12 Intelligent Lighting Institute, Eindhoven University of Technology, Eindhoven, the Netherlands; 13 Chronobiology, Faculty of Health and Medical Sciences, University of Surrey, Guildford, United Kingdom; 14 Translational Sensory & Circadian Neuroscience, Max Planck Institute for Biological Cybernetics, Tübingen, Germany; 15 TUM Department of Sport and Health Sciences (TUM SG), Technical University of Munich, Munich, Germany; 16 Department of Experimental Psychology, University of Oxford, Oxford, United Kingdom; 17 Circadian and Sleep Epidemiology Laboratory, Department of Integrative Physiology, University of Colorado Boulder, Boulder, Colorado, United States of America; 18 Department of Neurology, Northwestern University, Chicago, Illinois, United States of America; 19 Center for Circadian and Sleep Medicine, Northwestern University, Chicago, Illinois, United States of America; 20 Sleep and Chronobiology Laboratory, Department of Integrative Physiology, University of Colorado Boulder, Boulder, Colorado, United States of America

## Abstract

Ocular light exposure has important influences on human health and well-being through modulation of circadian rhythms and sleep, as well as neuroendocrine and cognitive functions. Prevailing patterns of light exposure do not optimally engage these actions for many individuals, but advances in our understanding of the underpinning mechanisms and emerging lighting technologies now present opportunities to adjust lighting to promote optimal physical and mental health and performance. A newly developed, international standard provides a SI-compliant way of quantifying the influence of light on the intrinsically photosensitive, melanopsin-expressing, retinal neurons that mediate these effects. The present report provides recommendations for lighting, based on an expert scientific consensus and expressed in an easily measured quantity (melanopic equivalent daylight illuminance (melaponic EDI)) defined within this standard. The recommendations are supported by detailed analysis of the sensitivity of human circadian, neuroendocrine, and alerting responses to ocular light and provide a straightforward framework to inform lighting design and practice.

## Abstract 2

Light exposure influences human health and wellbeing by modulating circadian rhythms and sleep. This Consensus View outlines the first expert scientific consensus recommendations for appropriate daily patterns of light exposure to promote health and wellbeing and inform lighting design and practice.

## Introduction

Besides supporting visual perception, ocular light exposure influences many aspects of human physiology and behaviour, including circadian rhythms, sleep, and alertness (both via circadian system–dependent and circadian system–independent routes), mood, neuroendocrine, and cognitive functions (reviewed in [1–4]). This array of retinally driven responses to light (collectively termed “non–image-forming” or, as used here for brevity, “nonvisual”) are important determinants of health, well-being, and performance, and some are already clinically relevant, as evidenced by current light therapy for circadian rhythm sleep disorders and various forms of depression [5–7]. Industrialisation and urbanisation have progressively and dramatically altered individuals’ light exposures, resulting in less light, including natural light, during the daytime and less darkness during the night, due to spending more time indoors where electric lighting provides the dominant source of illumination. Substantial evidence indicates that such altered light exposure patterns (and associated circadian/sleep disruption) contribute to negative impacts on health, sleep, and productivity, ranging from acute increases in accident risk to increased incidence of cardiometabolic disorders and forms of cancer (reviewed in [8–14]). Therefore, there is an urgent need for evidence-led recommendations to help inform the design and application of light emission technologies and human light exposures.

To date, a key challenge when optimising light exposure for promoting human health, well-being, and performance has been the lack of an accepted scientific framework upon which to quantify the propensity for light to elicit the relevant responses and from which to base recommendations for lighting design and practice. Fortunately, as a result of several decades of scientific advances, research-based recommendations are now possible.

**Fig 1:**
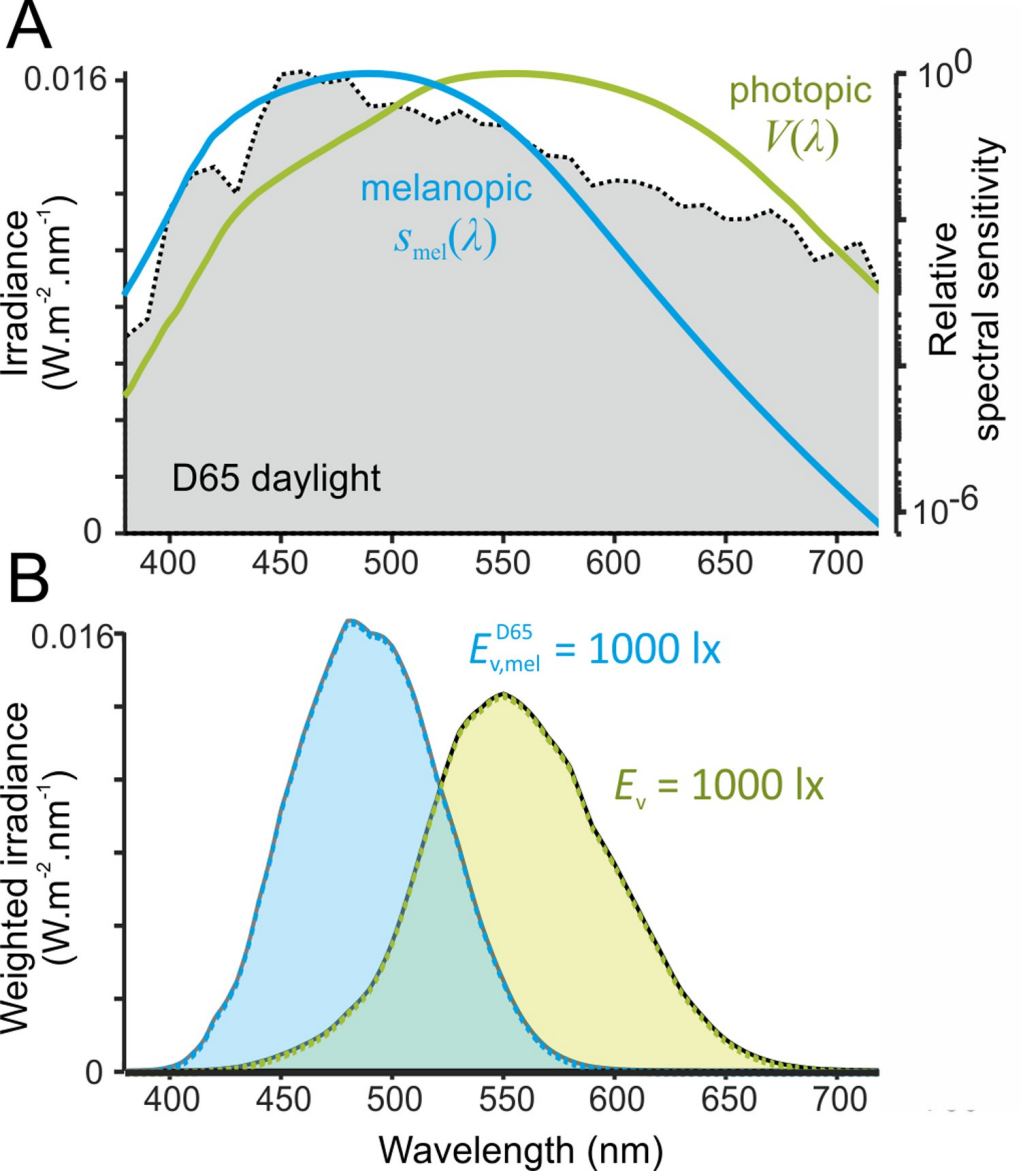
Differences in photopic and melanopic spectral sensitivity formalised in the SI-compliant system for quantifying ipRGC-influenced responses to light. Panel **A** illustrates the melanopic action spectrum (*s*_mel_(*λ*) with peak sensitivity at 490 nm, following prereceptoral filtering appropriate for a 32-year-old observer) and the photopic (2° spectral luminous efficiency) function, *V*(*λ*), superimposed on the spectral power distribution of standard daylight (CIE illuminant D65 [142]). Spectral sensitivities are plotted in logarithmic coordinates. Panel **B** illustrates the weighted spectral power distribution for spectrum in **A** multiplied by the photopic and melanopic efficiency functions at 1,000 lux for illuminance (*E*_v_) and melanopic EDI ($E_{v,mel}^{D65}$). Sensitivity curves in A are plotted from the tabulated values provided in the CIE S026 standard [34], with weighted irradiance (plots in B and associated calculations) derived using the procedures described in detail therein. CIE, Commission Internationale de l’Eclairage; ipRGC, intrinsically photosensitive retinal ganglion cell; melaponic EDI, melanopic equivalent daylight illuminance.

Building on initial observations that physiological responses to ocular light exposure can persist even in people who are totally visually blind [15–17], convergent evidence from studies of humans and animals has shown that such nonvisual responses (including effects on the circadian system, melatonin secretion, sleep/alertness, and pupil constriction) originate via a specialised class of retinal neurons, the intrinsically photosensitive retinal ganglion cells (ipRGCs) [18–26]. The light-sensing photopigment within the ipRGCs is melanopsin, which, in humans, is maximally sensitive to photons in a distinct portion of the visible spectrum to the cone photopigments (*λ*_max_ ≈ 480 nm before accounting for filtering through the lens and ocular media) [23,25,27]. As a result, the established photometric quantities used to describe brightness and luminous sensation as perceived by humans do not adequately reflect the spectral sensitivity of any melanopsin-dependent responses to light. Measures such as photopic (il)luminance, which primarily reflect the spectral sensitivity of long and medium wavelength sensitive cones, place substantially greater weight on longer wavelengths than those to which melanopsin is most sensitive. These measures therefore provide an inappropriate surrogate for quantifying the propensity of light to engage ipRGC-driven circadian, neuroendocrine, and neurobehavioural responses (Fig 1A).

While the potential value of a melanopsin-based photometric quantity has been recognised for some time, there has also been uncertainty as to whether this provides a sufficiently detailed model of the spectral sensitivity of human ipRGC-driven responses to ocular light exposure [28]. Hence, while the spectral sensitivity of physiological responses to light in visually blind people and animals matches that expected for melanopsin [20,23,25,29], in the fully intact retina, ipRGCs can also receive signals originating from rods and/or cones [26]. Moreover, available data indicate that the relative contributions of melanopsin and rod/cone photoreception to nonvisual ocular light responses, and consequently their apparent sensitivity, may vary as a function of exposure duration, light intensity, and perhaps time of day and/or prior light exposure [25,28,30–33].

As an initial response to the absence of a suitable metric for quantifying ipRGC-dependent ocular light responses, in 2013, an expert working group proposed a system that weighted irradiance according to the effective in vivo spectral sensitivity of the 5 known human retinal opsin proteins (melanopsin, rhodopsin, S-, M-, and L-cone opsin) [28]. This framework has now been formalised into an international standard with a SI-compliant system of metrology for ipRGC-influenced responses to light (Commission Internationale de l’Eclairage (CIE) S 026 [34]). Within this system, the effective rates of photon capture for each of the human retinal opsins under a given light condition are equated to the photopic properties (e.g., illuminance) of a standard 6500 K (D65) daylight spectrum that would produce the same rate of photon capture. This approach defines, for each opsin class, the α-opic equivalent daylight illuminance (EDI; where α-opic denotes one of the 5 human opsin classes that can contribute to ipRGC-influenced responses, e.g., melanopic; Fig 1B). Despite the significant advance provided by this new light measurement standard, to date, explicit scientific consensus guidance on the relationship between the 5 α-opic quantities and the magnitude of practically relevant ipRGC-dependent responses is lacking. For example, how should signals from melanopsin, cones, and rods be weighted? Do these weightings change with light exposure duration and history? What levels of α-opic EDI are appropriate in a given time of day and setting?

**Fig 2:**
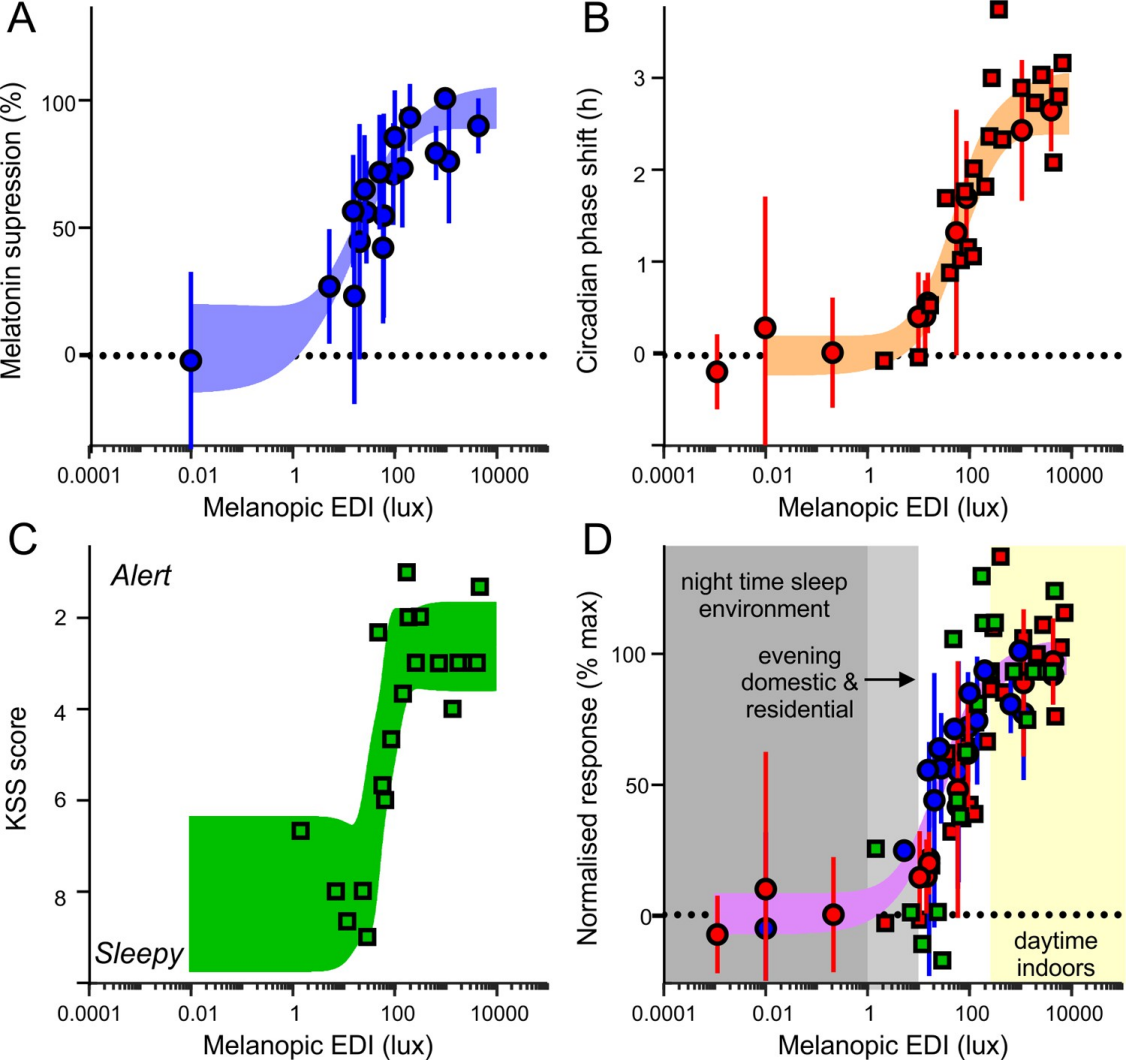
Recommendations for melanopic light exposures in relation to the sensitivity of melatonin suppression, circadian phase resetting, and subjective alerting responses. Data are derived from laboratory studies (in humans without the use of pupil dilators) investigating the impact of long exposures (>2 hours) to primarily broadband light sources on melatonin suppression [69,88,95,143,144] (**A**), circadian phase resetting [83,89,143,144] (**B**), and subjective alerting responses [86] (**C**), as analysed in [38]. Group data (round symbols) are presented as mean ± SD; otherwise, data for individual subjects are presented (square symbols). Shading represents the 95% confidence limits of an unconstrained 4-parameter sigmoid fit to the data. For comparison across different response types (**D**), data sets from **A**–**C** were normalised relative to the range of the curve fit for that response type. Shaded areas in **D** reflect the consensus recommendations of the Second International Workshop on Circadian and Neurophysiological Photometry for sleep, evening, and indoor daytime environments. Recommendations are intended to provide realistic targets that minimise inappropriate nonvisual responses in the sleep environment (melanopic EDI <1 lux) and reduce these so far as is practically possible presleep (3 hours before habitual sleep; melanopic EDI <10 lux) while maximising relevant effects across the intervening daytime hours (melanopic EDI >250 lux). The nonshaded region indicates the range of melanopic EDI that should, where possible, be avoided during evening and nighttime and are considered suboptimal for daytime environments. EDI, equivalent daylight illuminance.

Importantly, as originally envisaged [28], adoption of the new measurement approach has facilitated a number of large-scale retrospective evaluations of historical data [35–39] and informed new hypothesis-driven investigations [40–43] on the photoreceptive physiology for circadian, neuroendocrine, and neurobehavioural responses in humans. In total, the evidence from such studies [35–43] supports the view that, under most practically relevant situations (extended exposures to polychromatic light in the absence of pharmacological pupil dilation), light sensitivity of human physiological responses can be reliably approximated by the α-opic irradiance for melanopsin or the corresponding EDI (melanopic EDI). Moreover, based on the consistency of melanopic irradiance–response relationships across studies [38], it is now possible to define realistic, evidence-based recommendations for light exposures that target nonvisual responses (Fig 2). Alongside the emergence of freely available tools to calculate the relevant metrics [44,45], there now exists an easily measured and internationally accepted SI-compliant system of metrology [34] to inform lighting design and associated policy.

Here, we describe expert consensus-based recommendations for daytime, evening, and nighttime light exposure, considerations associated with their applicability, the supporting scientific evidence, and any caveats associated with the recommendations as they stand.

## Methodology

The Second International Workshop on Circadian and Neurophysiological Photometry in 2019 brought together experts in lighting, neurophysiological photometry and sleep, and circadian research (all workshop participants are included as authors of this manuscript). The workshop was chaired by Brown and Wright who invited participants based on professional and/or academic qualifications and on reputation of being a leading expert in the field, including being an author of key scientific publications and/or international standards on the topic. Workshop participants were provided goals and key questions to address prior to a structured face-to-face meeting. The primary focus of the meeting was to develop expert consensus recommendations for healthy daytime and evening/nighttime light environments tentatively based on the new SI-compliant measurement system (CIE S 026:2018) [34]. Initial questions for review and discussion were the following:

1. What range of melanopic EDI can be reasonably considered to provide minimal and maximal impacts on nonvisual ocular light responses in humans?
2. Do signals from rods and/or cones also play a major role, and, if so, what relevant guideline levels could be recommended to account for such actions?
3. Do the answers to (1) and/or (2) vary across different nonvisual forming responses (e.g., circadian entrainment/resetting, sleep/arousal, effects on hormone secretion, and mood) and, if so, what is the most appropriate general recommendation that can be provided?

Participants were also asked to consider if recommended light exposures would vary depending on which specific biological effects one is trying to achieve and/or on the target population (e.g., shift workers, specific clinical applications, etc.) and to include empirical literature supporting their views. In the face-to-face meeting, the morning of the first day was devoted to detailed presentations and discussion of the relevant scientific literature, and the afternoon was devoted to breakout sessions for discussion of questions 1 to 3 noted above. The second day was devoted to further discussion with sufficient time to address all opinions, ideas, and concerns. Voting to determine the expert consensus recommendations occurred via an iterative process; voting was limited to workshop participants and, where consensus could not be initially reached, discussion and review of the relevant literature resumed until participants were in agreement. Following the establishment of the expert consensus recommendations, a writing plan was formulated to produce the current paper. Subgroups of workshop participants initially drafted sections of the manuscript most relevant to their specialist expertise, including providing accounts of the scientific evidence from laboratory and field studies, relevance to other existing visual standards, and other special considerations associated with application of the recommendations. The workshop chairs (Brown and Wright) then integrated the expert content into a complete draft manuscript, including the recommendations formalised during the meeting. Workshop participants reviewed, edited, and approved both the draft (available as a preprint [46]), and this final version, which provides additional rationale supporting the recommendations and their practical application. The recommendations and associated considerations described herein are therefore the product of a workshop involving the authors. We are aware that such a workshop can rarely be exhaustive with respect to expertise and/or views across all potential stakeholders.

## Expert consensus-based recommendations

The recommendations, described below, are intended to provide realistic targets that will result in appropriate circadian, neuroendocrine, and neurobehavioural responses to ocular light exposure in humans.

### Daytime light recommendations for indoor environments

Throughout the daytime, the recommended minimum melanopic EDI is 250 lux at the eye measured in the vertical plane at approximately 1.2 m height (i.e., vertical illuminance at eye level when seated). If available, daylight should be used in the first instance to meet these levels. If additional electric lighting is required, the polychromatic white light should ideally have a spectrum that, like natural daylight, is enriched in shorter wavelengths close to the peak of the melanopic action spectrum (Fig 1A).

### Evening light recommendations for residential and other indoor environments

During the evening, starting at least 3 hours before bedtime, the recommended maximum melanopic EDI is 10 lux measured at the eye in the vertical plane approximately 1.2 m height. To help achieve this, where possible, the white light should have a spectrum depleted in short wavelengths close to the peak of the melanopic action spectrum.

### Nighttime light recommendations for the sleep environment

The sleep environment should be as dark as possible. The recommended maximum ambient melanopic EDI is 1 lux measured at the eye.

In case certain activities during the nighttime require vision, the recommended maximum melanopic EDI is 10 lux measured at the eye in the vertical plane at approximately 1.2 m height.

### Additional considerations

- Exposure to a stable and regular daily light–dark cycle is also likely to reinforce good alignment of circadian rhythms, which may further benefit sleep, cognition, and health. These recommendations should therefore be applied at the same time each day, so far as possible.
- These recommendations are not intended to supersede existing guidelines relating to visual function and safety. The nonvisual ocular light responses covered here should be an additional level of consideration provided that relevant visual standards can still be met.
- These recommendations are derived based on data from (and intended to apply to) healthy adults (aged 18 to 55) with regular daytime schedules. Special considerations may apply to specific populations (e.g., children, older people, shift workers, or other individuals whose light sensitivity deviates substantially from an “average” healthy adult) as discussed later in this publication (see “Special cases and exceptions”).

## Relationship to existing standards

There are several national and international standards that are relevant to indoor light exposure in the built environment, which have been developed under rigorous due processes, consensus, and other criteria. In terms of biological safety, there is a recent recommended practice for photobiological safety that provides guidance on ocular and dermal health relative to light exposure from all varieties of indoor lamps and lamp systems (American National Standards Institute/Illuminating Engineering Society (ANSI/IES) RP-27-20) [47]. The International Commission on Non-Ionizing Radiation Protection (ICNIRP) has also released a recent statement concerning photobiological safety, specifically of light exposure from LEDs [48]. Other existing guidelines, codes, and specifications for lighting installations in indoor places primarily concentrate on visual function, including visual comfort, visual performance, and seeing safely for people with normal, or corrected to normal, vision.

**Fig 3:**
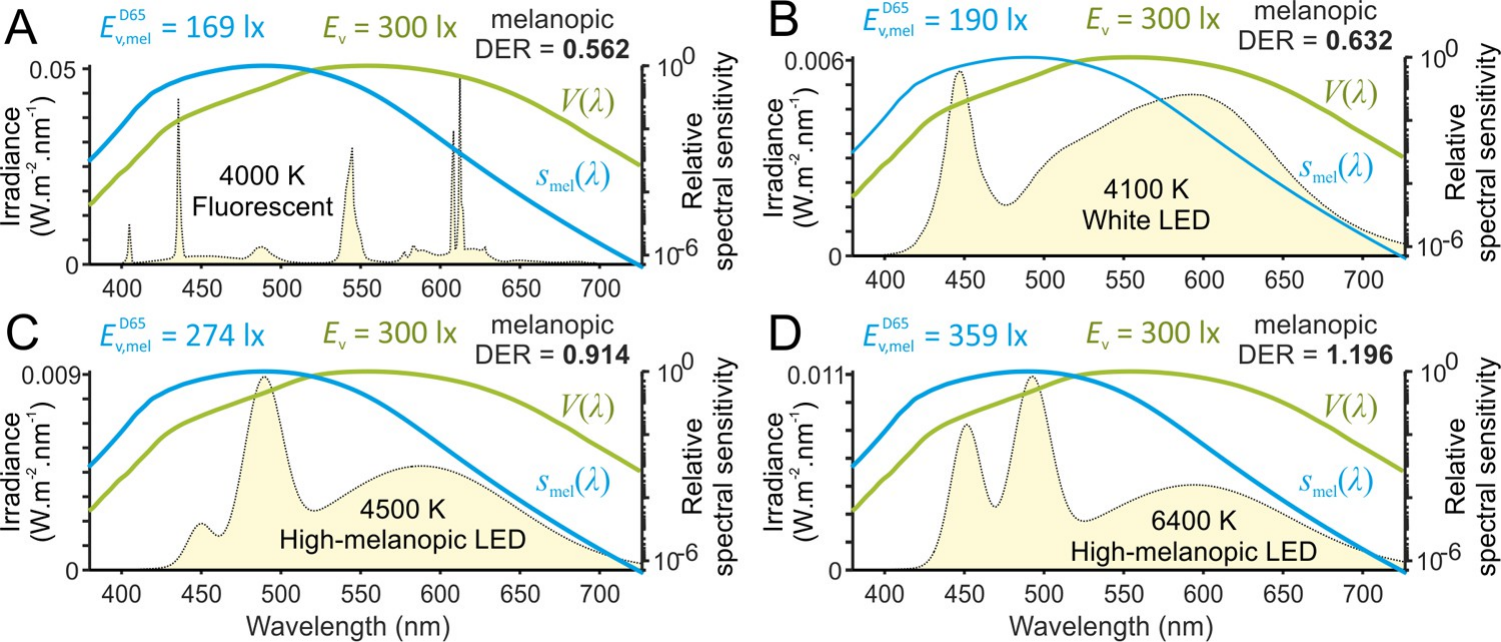
Impact of divergent spectral composition of electrical white light sources on melanopic efficiency. Panels **A** and **B** illustrate spectral power distributions (yellow) for commonly encountered fluorescent (A) and LED-based (B) white light sources. Panels **C** and **D** represent high melanopic content LED source of similar (**C**) and cooler (higher) correlated colour temperature (**D**) achievable with current technologies. Spectra in **A** and **B** represent CIE standard illuminants F11 and LED-B1, plotted from tabulated source data [142], spectra in **C** and **D** were modelled by combining weighted combinations of spectra from commercially available broad and narrowband LED sources. Melanopic (blue; *V*(*λ*)) and photopic (green; *V*(*λ*)) spectral efficiency functions are shown for reference. Photopic illuminance (*E*_v_) and melanopic equivalent daylight (D65) illuminance ($E_{v,mel}^{D65}$) for each spectrum is provided above, along with the melanopic efficiency for that light source (melanopic daylight (D65) efficacy ratio; melanopic DER, defined as the ratio of melanopic irradiances for this source to that for a D65 light source at the same photopic illuminance [34,52]). Note, in this example, all sources provide a photopic illuminance of 300 lux, but vary in melanopic EDI, due to the relatively low melanopic DER of commonly used white light sources. CIE, Commission Internationale de l’Eclairage; melanopic DER, melanopic daylight efficacy ratio; melaponic EDI, melanopic equivalent daylight illuminance.

Current specifications within lighting practice are based on illuminance and several additional qualitative and quantitative needs concerning glare, colour rendering, flicker and temporal light modulation, luminance distribution, and the directionality and variability (of both colour and level) of light. These specifications are crafted to enable people to perform their visual tasks accurately and efficiently, even for difficult circumstances or extended durations (e.g., Deutsches Institut für Normung (DIN) SPEC 67600 [49]; ANSI/IES RP-28-16 [50]; and EN 12464–1 [51]). Together with the focus on energy saving, the existing guidelines restrict the illuminance indoors to levels that are typically at least 1 order of magnitude below the natural light environment outdoors. Moreover, the electrical light sources in most common use, while optimised for their visual qualities, are typically substantially less efficient at stimulating melanopsin than natural daylight of equivalent illuminance, i.e., the light they provide has a low ratio of melanopic EDI to photopic illuminance (quantified by the melanopic daylight efficacy ratio (melanopic DER) [34,52]; see Fig 3). This leaves us with an indoor light environment that is potentially suboptimal for supporting human health, performance, and well-being [9–12, 53]. For example, Comité Européen de Normalisation (CEN) guidelines specify a minimum task plane photopic illuminance of 500 lux for writing, typing, reading, and data processing tasks. When just meeting this illuminance threshold with regular lighting (i.e., melanopic DER well below 1; Fig 3A and 3B), typical (vertical) melanopic EDIs encountered across the working day will fall below 200 lux (e.g., [54–56]). Moreover, specified illuminance levels for other settings, where visual demands are lower (e.g., corridors, rest rooms, etc.), will typically be substantially lower than the above (melanopic EDI <200 lux; [51]).

This publication is centrally based on an internationally balloted standard from the CIE [34], which now provides an accepted framework upon which to derive lighting specifications that optimise visual, circadian, neuroendocrine, and neurobehavioural responses to light. The corresponding expert-led consensus recommendations for biologically appropriate lighting are reflected in general melanopic EDI thresholds for various times of day/night. The recommendations presented here are intended to be widely achievable within the constraints of other relevant lighting guidelines (e.g., via lighting of appropriate spectral composition; Fig 3C and 3D) and to provide a sound scientific basis for the formal development of recommended practices in light and lighting from national and international standards organisations (e.g., ANSI, CIE, DIN, IES, and the International Organization for Standardization; ISO).

In closing this section, we note that a number of other recommendations relevant to physiological and neurobehavioural effects of light have been proposed in recent years, including some guidelines and specifications by commercial (for-profit) entities (reviewed in [57]). Unlike these previous suggestions, the present recommendations are both built around an SI-complaint, internationally accepted and validated measurement system and are supported by expert scientific consensus, features recognised as critical by established industry regulatory and standardisation bodies [58,59].

## Practical considerations

As noted above, while the recommendations detailed here are expected to be widely achievable, implementing these in any real-world setting necessitates care not to compromise other important regulations and/or considerations (e.g., visual appearance, glare, thermal comfort, safety, and energy efficiency). For example, an important consideration in achieving our recommendations for daytime settings is whether this would necessitate higher overall light levels and therefore increase energy expenditure and/or the risk of visual discomfort (e.g., glare). Notably, there is a range of approaches that (individually or in combination) could allow these recommendations to be met while avoiding such issues, including increasing the availability and accessibility of natural daylight (e.g., [56]), engineering the spectral content of electric lighting to increase melanopic DER, adjusting finishes and furnishings to optimise surface reflectances, and adjusting the placement, angular dispersion and size of accessible luminous surfaces to enhance vertical illuminances and/or minimise glare [60–64].

As an illustration of the above, a recent study modelling common office and educational settings found a combination of adjustments in surface finishes and spectral composition of lighting could readily produce 2- to 3-fold changes in vertical melanopsin-weighted light exposure [62]. For the conditions assessed there, where relevant national standards specified horizontal illuminances of 300 to 400 lux, achieving an average melanopic EDI of 250 lux in the vertical plane required an approximately 50% increase in horizontal illuminance and energy output when using an ‘off-the shelf’ tuneable white LED (6,500 K; melanopic DER = 0.83). With appropriate design, however, colour mixed LEDs can allow much higher melanopic DERs, even when maintaining warmer colour temperatures (e.g., up to 1.4 for 4,000 K sources [61]). Moreover, engineered LED luminaires that balance less extreme increases in melanopic DER with a good fidelity colour rendition index and slightly cooler white light [65] can facilitate meeting our daytime recommendations without significantly compromising energy expenditure or visual qualities (e.g., Fig 3D). Further, the energy efficiency of colour-mixed LED sources is rapidly approaching that typical of standard phosphor-converted LEDs and is scheduled to exceed this over the next decade [66]. Thus, while optimising building and lighting design to maximise energy efficiency and minimise visual discomfort remain important goals, these should not ultimately prove impediments to implementing our recommendations in most settings.

By contrast, increased energy expenditure is not a concern with respect to our evening/nighttime recommendations, where the significant practical consideration is rather ensuring that there is sufficient light to comfortably and safely perform visually guided activities. For the sleep environment, it is already natural to greatly minimise light exposure (by turning off lights, covering windows, and the like). There is likely still a fair proportion of individuals for whom the sleep environment is currently slightly above a melanopic EDI of 1 lux (e.g., [67 68]), although we would not envisage any significant barriers to reducing this where required (e.g., via use of blackout blinds and the installation of orientation lighting where needed). Outside of the sleep environment, however, ensuring sufficient light is available for vision is of course essential.

From existing ambulatory field assessments, evening (photopic) illuminance is commonly reported in the order of 30 lux [69–73]. Although certain rooms (e.g., kitchens) may be more brightly lit, this value of 30 lux corresponds to vertical illuminances typically measured in most indoor domestic environments in the evening [74]. In such cases, meeting the threshold melanopic EDI of 10 lux need not require any significant change in overall illuminance. Hence, many commonly used domestic warm white (2,700 to 3,000 K) LEDs already have a melanopic DER sufficiently low (<0.35 [52]) to meet our target while maintaining an illuminance of approximately 30 lux. Consistent with this view, a recent study that assessed evening light exposure in home settings via wearable spectrophotometers found that in nearly 50% of occasions melanopic EDI was already at or below 10 lux [75]. Moreover, many cases where evening light exposure was above this level involved lighting enriched in shorter wavelengths and could, in principle, been brought in line with our recommendations simply by using lower melanopic DER light sources. Further, the use of appropriate task lighting and/or lighting specifically engineered to minimise melanopic output (e.g., [69]) may further support activities that benefit from illuminances above 30 lux while maintaining an overall environment where melanopic EDI at the eyes remains below 10 lux (although the latter ultra-low melanopic DER sources will likely come at the expense of reduced colour discrimination). A particular challenge, however, comes from indoor environments outside the home (e.g., spaces shared by individuals with radically different daily/work schedules), where existing visual standards will often specify illuminance levels (>100 lux) that cannot be achieved while maintaining a vertical melanopic EDI <10 lux and optimal colour discrimination. Nonetheless, while meeting our evening target may not be achievable in all instances, it should be broadly achievable in most domestic settings with currently available lighting technology and, therefore, for those with the regular daytime work schedules for which it is intended to apply.

A final point for consideration relates to the likely benefits of implementing our recommendations that may have to be weighed up to justify any associated costs (e.g., due to upgrading workplace lighting). As discussed in detail below, there is certainly evidence that increased daytime light can improve subjective or objective measures of performance, sleep, alertness, and/or mood and that decreased evening and nighttime exposures can reduce adverse effects of light on sleep, circadian rhythms, and long-term health (see “Evidence from real-world settings”). Directly quantifying the benefits that might be expected associated with implementing our recommendations is far more challenging. In the future, large-scale longitudinal studies that combine data on objectively measured performance (e.g., sick days, productivity, and incidence of accidents), health outcomes, and appropriately measured (personal) light exposure may provide such information. In the interim, it is worth noting that, even ignoring possible impacts on the incidence of common and costly health complications associated with circadian disruption (e.g., cardiovascular disease, diabetes, and cancer), benefits associated with improved sleep alone could potentially be substantial [76]. Indeed, insufficient sleep is estimated to cost the United States 2.4% GDP, due absenteeism, accidents, reduced productivity, etc. [76,77]. Moreover, even comparatively modest improvements for those with poor sleep (<6 hours sleep/night increased to 6 to 7 hours/night) are predicted to increase US GDP in the order 1.7% or approximately 300 billion USD/year [77].

## Scientific rationale

### Evidence from laboratory studies

The rationale for basing these recommendations upon melanopic EDI is, in the first instance, provided by a comprehensive analysis of data aggregated from controlled laboratory studies (performed in healthy adults aged 18 to 55) that have evaluated the 2 best understood neuroendocrine and circadian light responses in humans: acute suppression of nocturnal pineal melatonin production and circadian phase resetting by evening or nighttime light exposure [36–39]. Those data indicate that, for a wide range of monochromatic, narrowband and broadband light sources and exposure durations, such ocular light responses are better predicted by melanopic irradiance than by photopic illuminance or other existing metrics. Additional contributions from photoreceptors other than melanopsin are expected based on known ipRGC biology [26,28], and evidence for such contributions has been observed under certain circumstances [30,78,79]. Importantly, however, the sum of empirical human data suggest that any such influences are sufficiently limited that, under most practically relevant circumstances, the spectral sensitivity of circadian and neuroendocrine and, by extension, other related nonvisual responses to ocular light exposure, can be well approximated by melanopic EDI.

The clearest evidence for contributions from photoreceptors other than melanopsin has so far come from evaluations of melatonin suppression in short (<1 hour) time windows following exposures to monochromatic light in participants with dilated pupils (to remove indirect effects of pupil constriction on apparent sensitivity). Data from 2 such studies are compatible with the possibilities that S-cones [78] or the photopic system [30] may contribute alongside melanopsin (see also reanalysis in [79]). Importantly, however, a large body of data with and without use of pupil dilation indicates that for exposures of an hour or more, melatonin suppression can be reliably predicted by melanopic EDI [37,38,80,81]. This conclusion is further strengthened by findings from recent studies that have employed photoreceptor isolating stimuli to confirm that melanopsin-selective changes in irradiance modulate melatonin production [40,41] but failed to find any effect of large variations in irradiance selectively targeting S-cones [42]. Further evidence consistent with a dominant role for melanopsin comes from earlier observations that totally blind humans (where remaining light responses match the spectral sensitivity expected for melanopsin) [23,25] can display near-full melatonin suppression [15,17,23], as do individuals with colour vision deficiencies [82].

In line with the data discussed above, totally blind individuals can also display circadian phase resetting responses to bright white light of comparable magnitude to sighted individuals [16]. Findings from one study in sighted individuals with pharmacologically dilated pupils are suggestive of cone contributions to circadian phase resetting following long (6.5 hours) exposures to dim monochromatic light [30]. However, an equivalent effect is not readily apparent across data from studies performed on participants with undilated pupils [38,83,84]. Thus, laboratory data collected under conditions that are more relevant to the real world, where pupils are freely light responsive, indicate that the influence of cones is sufficiently small that melanopic irradiance can provide a reliable approximation of the spectral sensitivity of circadian phase resetting.

By contrast to the circadian and neuroendocrine responses discussed above, other relevant effects of light that are of importance but mechanistically less well understood, such as acute light effects on alertness, have not yet received the same degree of analytic and parametric study. Nonetheless, light-dependent changes in subjective alertness have commonly been reported (reviewed in [2,85]) and, where performed, functional studies employing electroencephalogram (EEG) or magnetic resonance imaging approaches reveal clear neurophysiological correlates of such subjectively measured alertness changes (e.g., [43,86,87]).

With respect to the conditions under which such alerting effects occur, a recent comprehensive meta-analysis reveals that self-reported alerting responses to white light are commonly observable within a similar range of light intensities to those associated with effects on the circadian system (irrespective of time of day) [2]. Many of the original studies contributing to the latter analysis predate the discovery of melanopsin. It is possible, however, to obtain reasonable approximations of melanopic EDI from the photopic illuminance reported by earlier studies, by reference to the typical ratio of these 2 parameters expected for the relevant light sources (i.e., melanopic DER). For example, a recent meta-analysis [2] notes a significant subjective alerting effect of bright white light in almost 80% of studies (15 of 19) where the “dim” light condition was below 80 lux and the “bright” condition >500 lux (values that correspond to melanopic EDI of <50 lux and >250 lux, using a conservative melanopic DER of 0.6 and 0.5, respectively). Further, the published irradiance response data for subjective (and objective) alerting responses to nocturnal broadband white light exposure [86] align very well with the relationship between melanopic EDI and circadian-related responses determined from other studies that did not employ pupil dilation [38,88,89] (Fig 2).

The recent meta-analysis discussed above [2], which could not reach definitive conclusions regarding spectral sensitivity of alerting responses, did not assess the extent to which the magnitude of alerting responses were predictable based on melanopic EDI. Nonetheless, the most informative studies included in that analysis [69,90–94] and other relevant studies and meta-analyses [36,38,39,69,95] indicate that alerting effects produced by light of varying spectral composition are certainly better predicted by melanopic irradiance than other available metrics. Moreover, recent studies provide evidence that selectively increasing melanopic irradiance, in the absence of changes in either illuminance or colour, can promote self-reported alertness during both day [43] and evening [40]. The former study also confirmed EEG correlates of enhanced daytime alertness via alpha attenuation test [43]. Collectively, these data do not exclude the possibility that cone signals might exert a greater influence over acute alerting responses to light than is apparent for circadian and neuroendocrine effects. Nonetheless, the bulk of available evidence supports the view that melanopic EDI is the best currently existing predictor of alerting responses to light and is relevant for both day and evening/nighttime scenarios. The currently available data do not provide any definitive evidence that the sensitivity of such alerting responses differs substantially relative to other melanopsin-driven responses to evening/nighttime light exposure (Fig 2) or between night and day (reviewed in [2,85]). Accordingly, in the absence of new information, the sensitivity range defined for the more comprehensively studied circadian and neuroendocrine responses can be used as a sensible predictor of propensity of light to modulate alertness, regardless of time of day.

In sum, most of the available laboratory data suggest that melanopic EDI is a reliable index that provides a good approximation of the apparent spectral sensitivity of human circadian and acute nonvisual responses to ocular light exposure. In particular, for the extended exposures to polychromatic light that are relevant to everyday living environments, existing evidence indicates that any additional contributions from cones (or rods; whose spectral sensitivity is close to melanopsin [34,96,97]) do not compromise the predictive value of melanopic EDI.

As befitting a system evolved to optimise physiology and behaviour in anticipation of day–night transitions driven by the Earth’s rotation relative to the sun, the operating range of human circadian, neuroendocrine, and alerting responses to ocular light exposure spans the range of light intensities typically encountered between civil twilight and sunrise/sunset (i.e., melanopic EDI of approximately 1 to 1,000 lux; Fig 2). The recommendations indicated above are therefore intended to ensure that the sleeping environment is kept at a limit below which any appreciable nonvisual responses of this nature are elicited and to minimise negative effects of the light environment during sleep and presleep hours [98]. Similarly, recommendations for daytime and evening light exposure are intended, so far as practically possible, to respectively maximise and minimise any associated effects on sleep, alertness and the circadian system. By providing an appropriately marked day–night signal and reducing potential disruptive effects of evening light, collectively, these recommendations are expected to promote robust and appropriately timed circadian functions in most individuals [99], as well as to promote alertness throughout the day and support healthy sleep.

Also worthy of note here, a number of studies have provided evidence that undesirable effects of evening/nighttime light can be mitigated by brighter light exposure earlier in the day (e.g., [31,33,100–104]). While opposing actions of light exposure during morning/daytime and evening are a well-understood feature of circadian function [105,106], these modulatory effects also extend to more acute actions of evening light, such as its ability to suppress melatonin production. At present, the physiology responsible for such actions are not well understood, nor does currently available data enable a detailed assessment of the intensity and/or time range across which such effects operate. What is clear, however, is that modulatory effects of prior light exposure are certainly not limited to earlier parts of the day [33,104]. Accordingly, such observations suggest a further potential benefit of maintaining high melanopic light exposure throughout the day. The visual requirements necessary or desirable for some activities during later parts of the evening (e.g., relating to illuminance and/or colour) place a limit on the extent to which disruptive effects of white light can be entirely avoided simply by reducing melanopic EDI (e.g., using lighting with a lower melanopic DER). Higher levels of daytime light exposure may therefore help mitigate any disruptive effects associated with unavoidable light exposure in later parts of the evening.

### Evidence from real-world settings

While our current understanding of the spectral sensitivity and dynamic range of circadian, neuroendocrine, and neurobehavioural light responses in humans is most directly informed by laboratory studies, our recommendations are also supported by field evaluations of the impact of environmental lighting.

Access to electric lighting is associated with reduced daytime and increased nighttime light exposure and altered sleep timing [107–110], with many individuals in modern society routinely experiencing melanopic EDI <250 lux during the day, especially those with delayed sleep schedules [72,73]. Accordingly, there have been a number of real-world studies implementing daytime high melanopic lighting interventions in workplaces, schools, and care homes that provide practical corroboration for the recommendation outlined above [111]. Such practically focused investigations were not designed to evaluate melanopsin contributions per se (with increases in melanopic EDI usually being accompanied by increases in colour temperature and/or illuminance), but such studies do provide valuable insight in likely real-world benefits associated with meeting our recommendations.

In offices, increasing the melanopic output of architectural lighting (approximately 2-fold) via short wavelength-enriched lamps (17,000 K; melanopic DER ≈1, versus 3,000 to 4,000 K; melanopic DER<0.6) had beneficial effects on self-reported alertness, performance, mood, and sleep quality [54,55]. Similarly, enhancing daytime melanopic exposure by increased access to natural daylight in the workplace improved sleep and objectively measured cognitive performance (higher-order decision-making) in office workers [56]. In these studies [54–56], the average melanopic EDI in the control working environment was <150 lux (standard 3,000 to 4,000 K fluorescent lighting; Fig 3A), with the experimental “active” conditions increasing melanopic EDI to approximately 170 to 290 lux. Hence, modest and readily achievable adjustments to increase light exposure can be associated with measurable benefits, without any observable detrimental effects.

In schools, findings from a series of studies employing fluorescent lighting with various intensities and spectra indicate that settings with a higher melanopic output (melanopic EDI >500 lux) can improve measures of concentration and reading comprehension compared to current standard lighting (typically providing melanopic EDI <200 lux; [112–115]). Similar benefits of short wavelength-enriched (17,000 K) versus standard 4000 K fluorescent light on reducing sleepiness have also been shown in college-aged students during afternoon lectures [116]. Further, building on seminal work showing the benefits of increased daytime light levels for the elderly [117], several clinical trials have shown the benefits of enhanced melanopic light exposure during daytime hours on care home residents [118–120]. There is evidence of reduced circadian sensitivity/responsiveness to light in older adults (see “Special cases and exceptions”), including changes in lens transmission that could reduce effective retinal dose corresponding to a given melanopic EDI by approximately 50% relative to the (young) standard observer on which calculations are based [44]. Nonetheless, in these studies, compared to control conditions (typical daytime melanopic EDI <150 lux), implementation of higher melanopic, short-wavelength enriched, polychromatic lighting (5,500 to 17,000 K, providing melanopic EDI >250 lx) led to a range of improvements including reduced depression, agitation, and anxiety, better daytime activity and, in some studies, improved sleep quality.

Collectively, increasing melanopic light exposure during the day in line with our recommendations has been shown to benefit alertness, performance, and sleep in a wide range of real-world settings, even in the presence of daylight or stimulants such as caffeine or for younger or older age groups. Further, there is minimal evidence for negative effects of increased daytime melanopic light exposures. One care home study [120], where the brightest daytime light intervention was examined (bright 17,000 K lighting providing melanopic EDI approximately 900 lux), reported a reduction in sleep efficiency and quality when compared to standard 4000 K lighting (melanopic EDI approximately 100 lux). Further, in an office study of dayworkers where the melanopic EDI of control condition was already high (approximately 400 lux) further increases (melanopic EDI approximately 750 lux) associated with the use of an 8000 K lighting system appeared to prevent the normal seasonal advance in sleep timing [121]. While the latter could be considered beneficial, as it enhances circadian alignment to the working day, long-term effects of decoupling from seasonal environmental rhythms is, to date, unclear. Given these data, future research is warranted to identify the potential beneficial and adverse effects on human physiology, cognition, behaviour, and health of electric lighting that greatly exceeds our intensity recommendations.

In addition to reduced daytime light exposure, increased exposure to electric light in the evening and night is commonly considered to exert adverse effects on sleep, circadian rhythms, and health outcomes [8–11,67,68,122]. Indeed, even relatively low levels of light in the sleep environment (conservatively, melanopic EDI >3 lux) have been associated with impaired sleep and increased incidence of diabetes in large cohort studies [67,68]. Further, typical evening light levels often fall within the range where significant nonvisual responses would be predicted from laboratory studies [75]. For example, a significant source of evening light exposure is from visual displays, which in the absence of any other illumination, can provide melanopic EDI of >60 lux [52,123,124] (above the typical level of exposure required to produce half-maximal subjective alerting, melatonin suppressing, and circadian phase shifting responses in laboratory studies; Fig 2). Indeed, several studies have shown that light from modern visual displays is sufficient to reduce the evening rise in melatonin, impair sleepiness, and/or increase subjectively or objectively measured alertness [123–126]. Moreover, manipulations that reduce exposure to short wavelength light from such displays has, in some laboratory studies, been found to lessen these effects [125,126] as have selective reductions in melanopic output [40]. There have not yet been large-scale longitudinal field studies on how effective such manipulations might be, although it is noteworthy that the reductions in melanopic radiance achievable simply by adjusting the spectral content of current visual displays are modest (approximately 50% decrease). As such, we expect that such approaches will be most beneficial when combined with other strategies to minimise evening illumination (e.g., dimming of screens and low melanopic ambient lighting). In addition, the potential protective role of adequate daytime light exposure to attenuate adverse effects of evening and nighttime light exposure on circadian physiology requires future research.

### Special cases and exceptions

While the current recommendations are intended to be widely applicable, the scientific underpinnings primarily derive from studies of neuroendocrine, circadian, sleep, and alerting responses to ocular light exposure in healthy young adults. Even among this group, findings from a recent laboratory study show significant (>10-fold) interindividual variations in sensitivity to white light–induced suppression of the evening rise in melatonin [88]. The physiology underlying this variability is currently unknown. Importantly, however, assuming such variability is indicative of that for the healthy adult populations contributing across the range of lab studies discussed above, it is inherently incorporated into our recommendations. Hence, targets for daytime and nighttime exposures are based on light intensities found to produce near maximal or minimal responses across the test population. With respect to the recommendation for evening settings, there may be more significant variability in the relative magnitude of circadian, neuroendocrine, and associated neurophysiological responses, based on the intraindividual differences noted above [88]. In the absence of any ready means for predicting individual differences in sensitivity, the present recommendation of a maximum melanopic EDI of 10 lux is intended to appropriately minimise undesirable effects of evening light for the “average” healthy adult while allowing for sufficient light for common evening activities (see “Practical considerations” for further discussion). As it stands, where the evening light environment currently results in a melanopic EDI above 10 lux, reducing exposure in line with our recommendations is certainly still expected to be beneficial, regardless of individual differences in sensitivity, although future developments may make it possible to refine recommendations for specific individuals.

The magnitude of circadian and neuroendocrine responses to light also depends on age, with those in young children being larger and those in older adults tending to be smaller when compared to young adults [127–131]. These observations may, in part, reflect age-related differences in the amount of light reaching the retina (due to changes in pupil size and lens transmittance), although more direct changes in sensitivity or amplitude may also be involved. Certainly, one previous study that investigated the impact of age-related changes in lens transmittance did not find that this was associated with the expected reduction in light induced melatonin suppression in older adults [132]. Changes in light exposure in line with the current recommendations are still expected to be of general benefit to both young [112–115,124,126] and older individuals [67,68,118–120] (where their current daytime light exposure falls below, or evening/nighttime exposure above, the relevant targets). Select groups, however, may further benefit from higher daytime (e.g., older people) and/or lower evening exposures (e.g., children) than indicated in the recommendations. Similarly, disruptions to sleep and circadian rhythms are commonly associated with many disorders and disease states [8,133]. While adjusting light exposure may be of benefit in some or all of these conditions, further research will be required to determine whether alterations to the recommended thresholds will be required for such individuals.

In addition to the points above, a particular challenge in optimising light exposure to benefit health and performance relates to shift workers. Current light exposure advice for night shift workers is still not mature [134], and we want to stress that the present recommendations are not intended for this purpose. There is certainly evidence that increasing melanopic light levels in the work environment can improve subjective and/or objectively measured alertness and performance in shift workers [135–138]. Important benefits such as these do, however, need to be weighed in the context of potential disruptions to circadian alignment and chronic effects on health [8–14]. Addressing these important questions remains a key area for future investigation and shift work–related consensus guidance on best practice.

As discussed above, it is also essential that any changes to light exposure intended to adjust melanopsin-dependent physiological responses do not compromise visual requirements. For example, the elderly may need brighter lighting than recommended above to move safely between the bedroom and bathroom at night [12]. In many cases, such issues may be addressed by using lighting with an appropriate spectral composition (i.e., by using lighting with a low melanopic DER) and/or lighting designs that avoid direct illumination of the eyes. Nonetheless, there may be some instances where meeting the requirements for visual performance, visual comfort, and safety are incompatible with our recommendations regarding nonvisual responses, in which case the former must take precedence. Finally, while it is possible to comply with the recommended melanopic EDI thresholds specified here solely via exposure to electric light, there are a number of known and suspected benefits of exposure to broad-spectrum, outdoor, daylight [53,139–141].

## Future directions

The recommendations outlined here are derived from a synthesis of several decades of research into the biology regulating circadian, sleep, physiological and cognitive responses to light and their practical implications. There is, without question, evidence that the use of melanopic irradiance as a model for the spectral sensitivity of such responses represents a simplification of the underlying biology. Although, as an aside, we note that this is true also for the established and widely used, photometric quantities (luminance and illuminance) that are currently applied to quantify conventional “brightness.” Nevertheless, we leave open the possibility that a deeper understanding of rod and/or cone contributions to physiological responses will reveal multiphotoreceptor models of spectral sensitivity that may allow a more accurate prediction of circadian, sleep, neuroendocrine, and/or cognitive responses. The contribution of rods to such responses is an interesting topic for research in its own right. Nonetheless, including a rod component in any such future metrics is unlikely to substantially improve the accuracy with which they recreate the spectral sensitivity of the relevant response(s), since the very similar spectral sensitivity profiles of rods and melanopsin render effective irradiance for these 2 opsins highly correlated [97]. Conversely, cone spectral sensitivity is quite distinct from melanopsin and has the potential to substantially refine metrics for circadian and neurophysiological responses. In particular, future work may reveal specific lighting conditions that maximise cone influence to produce practically relevant modulations in nonvisual responses to light (e.g., on the circadian system, neuroendocrine function, sleep physiology, and/or and alerting responses). At present, however, existing evidence indicates that the use of melanopic irradiance/EDI would not lead one to substantially over- or underestimate biological and behavioural effects for the types of light exposure that are typically encountered across daily life [35–38,40–43].

Further research into the factors influencing individual differences in the sensitivity of melanopsin-mediated responses to light exposure may make it possible to tailor guidelines to specific groups or even individuals. For the time being, our recommendations are derived from group data that must incorporate much of this variability. As such, it is expected that the recommendations for daytime and the sleep environment should be broadly applicable and strongly engage relevant circadian and neurophysiological responses for the vast majority of healthy adults. Known, age-related sources of variability are already at least partly accounted for by the inclusion of corrections for changes in lens transmission described in the nonnormative appendices of the existing standard [34]. Recommendations may, however, be modified in the future for certain groups such as children, older adults, or patient groups whose sensitivity to light may differ from the healthy adult population on which the present recommendations are based.

The current recommendations are intended to inform lighting design considerations for typical, real-world environments such as offices and other workplaces, schools, and colleges, residences, care homes, and in- and outpatient settings. As noted above, application of our recommendations across such settings is facilitated by the free availability of tools for calculating melanopic EDI (and also estimating this given known illuminance and type of lighting) [44,45]. Nonetheless, the emergence of low-cost commercial sensors for direct measurement of melanopic EDI (akin to conventional “lux meters”) is expected to further increase the ease with which the recommendations can be adopted.

A final point for consideration relates to applications of light therapy for clinical conditions like affective and circadian rhythm sleep disorders or for purposes such as improving circadian regulation and alertness in night and shift workers or transmeridian travellers experiencing jet lag. The current recommendations are not directly formulated for such uses, but the existing applications of ocular light therapy likely involve the same or similar biological underpinnings as discussed above. Given existing evidence for benefits of bright light therapy [5–7], perhaps widespread adoption of the recommendations described here will contribute to a reduction in the prevalence of affective and sleep disorders. More significantly, however, we expect the scientific framework that informs these recommendations to provide a concrete basis upon which to generate hypotheses to test for the subsequent establishment of optimal light treatment recommendations for clinical and travel applications.

## Abbreviations

ANSI: American National Standards Institute
CEN: Comité Européen de Normalisation
CIE: Commission Internationale de l’Eclairage
DIN: Deutsches Institut für Normung
EEG: electroencephalogram
ICNIRP: International Commission on Non-Ionizing Radiation Protection
IES: Illuminating Engineering Society
ipRGC: intrinsically photosensitive retinal ganglion cell
ISO: International Organization for Standardization
melanopic DER: melanopic daylight efficacy ratio
melaponic EDI: melanopic equivalent daylight illuminance
